# Anesthetic Agents as Modulators of Antitumor Immunity: Repurposing Etomidate for Hepatocellular Carcinoma Therapy

**DOI:** 10.1016/j.jcmgh.2025.101650

**Published:** 2025-09-29

**Authors:** Hanchao Lin, Qiongzhu Dong

**Affiliations:** Key Laboratory of Whole-Period Monitoring and Precise Intervention of Digestive Cancer, Shanghai Municipal Health Commission, Minhang Hospital, Fudan University, Shanghai, China

Hepatocellular carcinoma (HCC) continues to be a highly aggressive cancer with a poor prognosis and limited effective treatment options.^1^ Although curative interventions like surgical resection, liver transplantation, and ablation are only feasible for a limited subset of patients, the treatment landscape for advanced HCC has expanded to include several molecular targeted agents (eg, sorafenib, lenvatinib, regorafenib, cabozantinib) and immunotherapies (eg, nivolumab and pembrolizumab).^2^ Recently, emerging evidence suggested that perioperative anesthetic agents may influence the long-term outcomes of patients with HCC through various mechanisms, extending beyond their primary roles in analgesia and sedation.^3^ This highlights their potential role as an adjunctive therapy to enhance treatment efficacy.

Etomidate, a widely utilized intravenous anesthetic, has been shown to suppress the intrinsic tumorigenic activity of HCC cells.^4^ In this issue of *Cellular and Molecular Gastroenterology and Hepatology*, Xu et al^5^ demonstrated that etomidate enhances antitumor immunity by suppressing programmed death-ligand 1 (PD-L1) expression in HCC cells. Their findings elucidate, for the first time, the molecular mechanism through which etomidate modulates the tumor immune microenvironment (TIME) in HCC. The authors demonstrate that etomidate markedly inhibits HCC progression and reveal a strong correlation between its potent antitumor effects and the immune response. Utilizing a combination of single-cell cytometry by time-of-flight and multiplexed immunohistochemistry, they find that etomidate treatment significantly improves the immunosuppressive microenvironment in HCC. In previous work, the authors’ team discovered that etomidate exerts antitumor effects by inhibiting the Janus kinase 2 (JAK2)/signal transducer and activator of transcription 3 (STAT3) signaling pathway in HCC.^4^ Building on their previous finding, the current study further reveals that etomidate inhibits the transcriptional activation of PD-L1 through suppression of the JAK2/STAT3 signaling axis, leading to its downregulation. This mechanism subsequently enhances the infiltration and activation of CD8^+^ T cells, thereby augmenting antitumor immunity. This is in accordance with previous findings that other commonly used clinical anesthetics, such as propofol and lidocaine, modulate host immune responses and influence tumor progression.^6^ Although previous studies have suggested a correlation between etomidate and certain immune responses,^7^ this study identifies the JAK2/STAT3/PD-L1 axis as the key mechanism by which it modulates the TIME in HCC.

Given that etomidate-induced suppression of PD-L1 expression enhances antitumor immunity, Xu et al further investigate its potential synergy with immune checkpoint inhibitors (ICIs). They demonstrate that etomidate significantly enhances the antitumor efficacy of anti-PD-L1 therapy, a finding that was robustly validated in both patient-derived organoid and patient-derived xenograft models. These collective findings position etomidate as a promising adjunctive agent to ICIs, with the potential to enhance the efficacy of immunotherapeutic strategies. Among the various therapeutic strategies that combine anti-PD-1/PD-L1 antibodies with other agents, the use of JAK/STAT inhibitors in combination with ICIs is regarded as a promising approach for cancer treatment.^8^ Currently, multiple clinical trials are assessing different JAK/STAT inhibitors in combination with anti-PD-1/PD-L1 antibodies. Given the regulatory effect of etomidate on the JAK2/STAT3 signaling pathway, this study may offer a novel and potentially effective therapeutic strategy for HCC.

Current evidence indicates that etomidate modulates multiple signaling pathways (such as NF-κB^9^ and PI3K/Akt^10^) that are known to directly or indirectly regulate PD-L1 expression in tumor cells.^11^ Therefore, the downregulation of PD-L1 by etomidate in HCC cells likely results from the synergy of multiple mechanisms. As we know, the TIME of HCC constitutes a highly heterogeneous system characterized by dynamic interactions among diverse immune cells, cytokines, and signaling pathways.^12^ Future studies should further elucidate the complex regulatory networks through which etomidate modulates immunosuppression in cancer. Such efforts should extend beyond CD8^+^ T cells to include a more comprehensive analysis of etomidate`s effects on the TIME, particularly focusing on macrophage, natural killer cell, and eosinophil subsets, for which preliminary evidence suggests potential modulation by etomidate.^7^ Furthermore, as an anesthetic agent, the use of etomidate in combination with immunotherapy requires careful timing within the perioperative period. It is essential to clarify the duration of its immunomodulatory effects on the TIME of HCC. Future studies should thoroughly validate critical factors such as the timing of administration, optimal dosing, and the combined safety of both the anesthetic and ICIs.

In summary, this study provides a preclinical rationale for the selection of anesthetic agents in the perioperative management of HCC, suggesting that etomidate may serve as a favorable anesthetic option to improve surgical outcomes, and further highlights its repurposing potential as an adjunct to immunotherapy. These results establish a theoretical foundation for the development of novel precision treatment strategies for HCC.
